# Semi‐automated Rasch analysis using in‐plus‐out‐of‐questionnaire log likelihood

**DOI:** 10.1111/bmsp.12218

**Published:** 2020-08-28

**Authors:** Feri Wijayanto, Karlien Mul, Perry Groot, Baziel G.M. van Engelen, Tom Heskes

**Affiliations:** ^1^ Department of Informatics Universitas Islam Indonesia Yogyakarta Indonesia; ^2^ Institute for Computing and Information Sciences Radboud University Nijmegen The Netherlands; ^3^ Department of Neurology Donders Institute for Brain Cognition, and Behaviour Radboud University Medical Center Nijmegen The Netherlands

**Keywords:** generalized partial credit model, penalized JMLE, rasch model, semi‐automated rasch analysis

## Abstract

Rasch analysis is a popular statistical tool for developing and validating instruments that aim to measure human performance, attitudes and perceptions. Despite the availability of various software packages, constructing a good instrument based on Rasch analysis is still considered to be a complex, labour‐intensive task, requiring human expertise and rather subjective judgements along the way. In this paper we propose a semi‐automated method for Rasch analysis based on first principles that reduces the need for human input. To this end, we introduce a novel criterion, called in‐plus‐out‐of‐questionnaire log likelihood (IPOQ‐LL). On artificial data sets, we confirm that optimization of IPOQ‐LL leads to the desired behaviour in the case of multi‐dimensional and inhomogeneous surveys. On three publicly available real‐world data sets, our method leads to instruments that are, for all practical purposes, indistinguishable from those obtained by Rasch analysis experts through a manual procedure.

## 1 Introduction

Our theoretical understanding of the world often contains latent constructs or traits, such as ‘intelligence’ and ‘quality of life’, that cannot be assessed directly. In an attempt to infer these traits for individual people, thousands of scales have been developed that aim to measure a range of behaviours and experiences in sociology, psychology, and medicine. These scales are typically based on questionnaires that contain a number of items. An appropriate instrument leads to test scores that are valid (measure the latent construct it has been designed to measure), reliable (consistently measure the observed latent construct), and clinimetrically useful. For practical purposes, we prefer questionnaires with a limited number of representative items, to lessen the burden on the respondents and increase the reliability.

One of the modern ways to develop a linear‐weighted, clinimetrically sound measurement instrument is Rasch analysis. The Rasch model can be used to create measurements from categorical or ordinal data, as a function of the trade‐off between the subject's abilities and the item difficulties. After conceptualization of the latent construct, a relatively large number of items are designed that are expected to relate to the trait under consideration. If available, an existing scale can be used instead. This original survey is provided to a number of initial respondents, preferably a few hundred in order to provide a stable Rasch model (Wright & Tennant, 1996). Their responses are then used for subsequent analysis. In this procedure, items are evaluated based on various criteria inherent in the Rasch model (Rasch, 1966, 1980), such as item goodness of fit, unidimensionality, presence of differential item functioning (DIF), and local dependency (Gustafsson, 1980; Hermans, Faber, De Baets, de Die‐Smulders, & Merkies, 2010). The item or items found to not fit the Rasch model are generally reworded, removed, or remodelled within the limits of the model (e.g., subset creation), after which the remaining items are reevaluated, until a clinimetrically optimal smaller itemset remains that fulfils all the model's expectations.

This step‐by‐step procedure is typically done manually, by expert Rasch analysts, with the help of software packages such as Winsteps, RUMM2030, ConQuest, or eRm.^1^ This procedure can be relatively time‐consuming, especially for large or complex datasets. Furthermore, since the decisions as to which items to include partly rely on human judgement and clinical expertise, different experts often arrive at different but equally suited instruments.

The aim of this paper is to try and automate the process of turning an ordinal scale into a clinimetrically sound linear‐weighted scale. We will show that our procedure naturally caters for typical Rasch criteria such as item goodness of fit, unidimensionality, and (to some extent) local dependency. We discuss extensions of our approach to also include DIF, restoring of disordered thresholds, and possibly other criteria, but leave actual implementations of these ideas for future work. We will refer to our procedure as ‘semi‐automated’, to acknowledge that it only automates part of a full‐blown Rasch analysis as well as to emphasize the need for sanity checks in any statistical analysis.

In Section 2 we discuss our central model, the generalized partial credit model (GPCM), its relation to other models and the idea of solving its estimation problem using regularization. Section 3 elaborates the vital part of our proposed method, the in‐plus‐out‐of‐questionnaire log likelihood (IPOQ‐LL), and argues for the method in comparison with the typical criteria of standard Rasch analysis. In this section we also explain our approach to avoiding exhaustive search on a survey with large numbers of items. Section 4 reports the results of the experiments on two artificial datasets (inhomogeneous and multi‐dimensional surveys) and three publicly available real‐world datasets. The R package containing the algorithm and results reported in this paper can be found on GitHub.^2^

## 2 Preliminaries

### 2.1 Generalized partial credit model

As explained in Section 1, in order to accomplish our aim we need to re‐evaluate our initial survey with P items, on which we have responses from N subjects, to a smaller and clinimetrically improved questionnaire. We consider the general case in which the responses are recorded in two or more ordered categories and write $x_{\mathrm{ni}}\in \left\{0,1,\ldots ,m_{i}\right\}$ for the observed response of subject n on item i, where item i consists of $m_{i}+1$ ordered categories. For binary responses (i.e., dichotomous test items) we simply set $m_{i}=1$. For polytomous test items we have $m_{i}>1$.

Rasch analysis relies on a mathematical, probabilistic model for the response of a subject on a particular item. In this paper we will consider the generalized partial credit model, introduced in (Muraki, 1992), a generalization of the partial credit model (PCM), which facilitates Rasch analysis of polytomous test cases. The GPCM contains different types of parameters. The parameter $\theta_{n}$ represents the *trait* or *ability* of subject n (the precise meaning of $\theta_{n}$ depends on the context, but we use both terms interchangeably to refer to the same thing): the higher the value of $\theta_{n}$, the greater the probability that subject n gives high responses. We refer to the parameter $\beta_{\mathrm{ij}}$, with $j=1,\ldots ,m_{i}$, as the *difficulty* or *threshold* of item i: the higher the value of $\beta_{\mathrm{ij}}$, the higher $\theta_{n}$ needs to be to make a response greater than j probable. In addition, and unlike most standard approaches in Rasch analysis, the GPCM includes so‐called *discrimination parameters* $\alpha_{i}$ that model the predictability of the responses on item i. These discrimination parameters are the reason why we use the GPCM. Furthermore, we will show that these parameters are closely related to the outfit and infit statistics in the Rasch analysis.

According to the GPCM, the probability that subject n gives response x on item i is given by.

(1) $$P\left(X_{\mathrm{ni}}=x|\theta ,\beta ,\alpha \right)=\frac{\exp\left[\alpha_{i}\sum_{j=1}^{x}\left(\theta_{n}‐\beta_{\mathrm{ij}}\right)\right]}{1+\sum_{k=1}^{m_{i}}\exp\left[\alpha_{i}\sum_{j=1}^{k}\left(\theta_{n}‐\beta_{\mathrm{ij}}\right)\right]},$$

for x>0, and.

(2) $$P\left(X_{\mathrm{ni}}=0|\theta ,\beta ,\alpha \right)=\frac{1}{1+\sum_{k=1}^{m_{i}}\exp\left[\alpha_{i}\sum_{j=1}^{k}\left(\theta_{n}‐\beta_{\mathrm{ij}}\right)\right]}.$$

With $\alpha_{i}=1$, for i=1,…,P, we obtain the (standard) PCM. In the dichotomous case, with $m_{i}=1$ for i=1,…,P, the GPCM simplifies to the two‐parameter logistic (2PL) model and the standard PCM further simplifies to the original Rasch model (Lord & Novick, 1968; Masters, 1982; Rasch, 1980).

### 2.2 Penalized joint maximum likelihood estimation

Given the observed responses $x_{\mathrm{ni}}$, we then define the log likelihood for a particular set of items $S\subset \left\{1,\ldots ,P\right\}$ as.

(3) $$L_{S}\left(\theta ,\beta ,\alpha \right)=\sum_{i\in S}\sum_{n=1}^{N}\log P\left(X=x_{\mathrm{ni}}|\theta ,\beta ,\alpha \right),$$

with $P(X=x_{\mathrm{ni}}|\theta ,\beta ,\alpha )$ from (1) and (2). This log likelihood aims to measure how well the parameters θ, β and α fit the subjects' observed responses on the items from set S.

In this paper we will apply (penalized) joint maximum likelihood estimation (van der Linden, 2016a; Wright & Douglas, 1975; Wright & Panchapakesan, 1969) whenever we aim to maximize this log likelihood. A well‐known drawback of maximum likelihood estimation, including the standard joint maximum likelihood estimation, is its lack of convergence, especially when dealing with a perfect score or a completely zero score (Bertoli‐Barsotti, 2005; van der Linden, 2016a). Furthermore, the GPCM is non‐identifiable: its outcome does not change when we add a constant to all abilities and thresholds, nor when we multiply all abilities and thresholds by a constant and divide the discrimination parameters by the same constant. To solve these identifiability issues, one can set the mean and variance of the θ to 0 and 1, respectively, or the parameters $\beta_{1}$ and $\alpha_{1}$ to 0 (van der Linden, 2016a, 2016b). Alternatively, one can implement the so‐called location constraint (Muraki, 1992). We choose to regularize the solutions by adding L2 (ridge) penalty terms and define the penalized log likelihood.

(4) $$F_{S\;}\left(\theta ,\;\;\beta ,\;\;\alpha \right)=L_{S\;}\left(\theta ,\;\;\beta ,\;\;\alpha \right)\;\;‐\;\;\lambda_{\theta }\sum_{n=1}^{N}\theta_{n}^{2}\;\;‐\;\;\lambda_{\alpha }\sum_{i\in S}(\ln\;\alpha_{i}){\;}^{2}.F_{S\;}\left(\theta ,\;\;\beta ,\;\;\alpha \right)=L_{S\;}\left(\theta ,\;\;\beta ,\;\;\alpha \right)\;\;‐\;\;\lambda_{\theta }\sum_{n=1}^{N}\theta_{n}^{2}\;\;‐\;\;\lambda_{\alpha }\sum_{i\in S}(\ln\;\alpha_{i}){\;}^{2}.$$

We set a penalty on the (natural) logarithm of $\alpha_{i}$ to drive these discrimination parameters towards 1. Values equal to 1 would correspond to the standard PCM that typically underlies polytomous Rasch analysis. Similar, but slightly different, penalties are considered by Paolino (2013) for the 2PL model and, more recently, by Chen, Li, and Zhang (2017) for the multidimensional item response theory model (Reckase, 2009).

## 3 The proposed method

### 3.1 In‐plus‐out‐of‐questionnaire log likelihood

When designing an instrument, we keep items which help us to construct a clinimetrically optimal scale and drop those that do not which represent the clinical picture as part of our experts' point of view. We will refer to the corresponding sets as the included itemset $S_{\text{in}}$ and the excluded itemset $S_{\mathrm{out}}=\left\{1,\ldots ,P\right\}\backslash S_{\mathrm{in}}$ (i.e., all items except for those that are part of $S_{\text{in}}$). Our aim is to come up with a single criterion for judging the quality of any split into $S_{\text{in}}$ and (thus) $S_{\text{out}}$ based on the observed responses on the original survey. Given such a criterion, one's favourite optimization procedure can be applied to search for the included itemset $S_{\text{in}}$ that maximizes it.

When we would indeed pick $S_{\text{in}}$ as our final questionnaire, we can only make use of a subject's responses on these items to estimate a subject's ability. To mimic this situation, we estimate the subjects' abilities that participated in the original survey only from their responses to $S_{\text{in}}$, for example, by maximizing the penalized log likelihood from (4):

(5) $$\left\{{\hat{\theta }}_{S_{\mathrm{in}}},\;{\hat{\beta }}_{S_{\mathrm{in}}},\;{\hat{\alpha }}_{S_{\mathrm{in}}}\right\}=\underset{\left\{\theta ,\beta ,\alpha \right\}}{\arg\max}L_{S_{\mathrm{in}}}\left(\theta ,\beta ,\alpha \right)‐\lambda_{\theta }\sum_{n=1}^{N}(\theta_{n}^{2}‐\lambda_{\mathrm{in}}\sum_{i\in S_{\mathrm{in}}}{\left(\ln\alpha_{i}\right)}^{2}.$$

We refer to the log likelihood.

(6) $$IQ‐LL\left(S_{\mathrm{in}}\right)=L_{S_{\mathrm{in}}}\left({\hat{\theta }}_{S_{\mathrm{in}}},{\hat{\beta }}_{S_{\mathrm{in}}},{\hat{\alpha }}_{S_{\mathrm{in}}}\right)$$

of these fitted parameters on the included itemset as the in‐questionnaire log likelihood. We would like the in‐questionnaire log likelihood to be relatively high, and indeed this is what typical test statistics in Rasch analysis like outfit and infit to some extent measure.

If, however, we trust that the designer of the original survey did a fine job and managed to come up with items that somehow relate to the latent construct that the final instrument is supposed to measure, the subjects' abilities ${\hat{\theta }}_{S_{\mathrm{in}}}$ should still provide a decent fit of the observed responses on the excluded set. To estimate the quality of this fit, we fix the abilities ${\hat{\theta }}_{S_{\mathrm{in}}}$ and only optimize over the thresholds and the discrimination parameters:

(7) $$\left\{{\hat{\beta }}_{S_{\mathrm{out}}},\;{\hat{\alpha }}_{S_{\mathrm{out}}}\right\}=\underset{\left\{\beta ,\alpha \right\}}{\arg\max}L_{S_{\mathrm{out}}}\left({\hat{\theta }}_{S_{\mathrm{in}}},\beta ,\alpha \right)‐\lambda_{\mathrm{out}}\sum_{i\in S_{\mathrm{out}}}{\left(\ln\alpha_{i}\right)}^{2}.$$

We refer to the resulting

(8) $$OQ‐LL\left(S_{\mathrm{out}}\right)=L_{S_{\mathrm{out}}}\left({\hat{\theta }}_{S_{\mathrm{in}}},{\hat{\beta }}_{S_{\mathrm{out}}},{\hat{\alpha }}_{S_{\mathrm{out}}}\right)$$

as the out‐of‐questionnaire log likelihood. Our final criterion, called the in‐plus‐out‐of‐questionnaire log likelihood (IPOQ‐LL), adds the two log likelihoods together:

$$IPOQ‐LL\left(S_{\text{in}},S_{\text{out}}\right)=IQ‐LL\left(S_{\text{in}}\right)+OQ‐LL\left(S_{\text{out}}\right).$$

Algorithm 1 summarizes the steps involved in the computation of the in‐plus‐out‐of‐questionnaire log likelihood. The IPOQ‐LL is nicely balanced in the sense that every item in the original full survey always contributes once, either to the in‐questionnaire or to the out‐of‐questionnaire log likelihood.

**Table:** 

**Algorithm 1** Recipe to compute the in‐plus‐out‐of‐questionnaire log likelihood for a particular included itemset $S_{\text{in}}$ and a corresponding excluded itemset $S_{\text{out}}$.
Fit abilities, thresholds, and discrimination parameters through penalized maximum likelihood estimation on the included itemset $S_{\text{in}}$ as in (5). Compute the in‐questionnaire log likelihood of the parameters obtained in step 1 on the included itemset $S_{\text{in}}$ as in (6). Fit thresholds and discrimination parameters with the abilities fixed to those obtained in step 1 through (weakly) penalized maximum likelihood estimation on the excluded items $S_{\text{out}}$ as in (7). Compute the out‐of‐questionnaire log likelihood of the thresholds and discrimination parameters obtained in step 3 and the abilities obtained in step 1 on the excluded itemset $S_{\text{out}}$ as in (8). Add the out‐of‐questionnaire log likelihood from step 4 to the in‐questionnaire log likelihood from step 2.

In all our experiments, we set $\lambda_{\theta }$ to .05 and $\lambda_{\text{in}}$ to 50 for the included itemset and $\lambda_{\text{out}}$ to .05 for the excluded itemset. Our procedure is largely insensitive to the precise setting of these parameters: just a little bit of regularization is sufficient to resolve issues with non‐convergence and non‐identifiability. The stronger regularization on the discrimination parameters in the included set and weaker (or even absent) regularization on the discrimination parameters in the excluded set is essential to get a better screening for an appropriate instrument.

### 3.2 Comparison to standard Rasch analysis

Standard Rasch analysis follows a manual iterative approach, in which items are evaluated and removed one by one if needed (Hermans *et al*., 2010, 2013; Van Nes *et al*., 2011). Typical evaluation criteria are item goodness of fit (Wright & Masters, 1982), unidimensionality (Hattie, 1985; G. H. Fischer & Molenaar, 1995) and local dependency (Christensen, Kreiner, & Mesbah, 2013). In the following, we will explain why we expect our procedure to naturally incorporate these criteria.

#### 3.2.1 Goodness of fit

A standard procedure in Rasch analysis is to remove items with infit and outfit values that are very different from 1, and in particular those that are much larger than 1 (indicating more noise than expected). Our procedure will have a tendency to put predictive items in the included itemset: they help to reliably estimate the abilities and hence to achieve a higher log likelihood, not only on the included items but also on the excluded items. Too predictable or too unpredictable items are more likely to end up in the excluded set, with more flexibility (less penalty) to estimate the discrimination parameter.

#### 3.2.2 Unidimensionality

Rasch analysis assumes that there is a single, unidimensional latent construct. Tests, such as the Martin–Löf test (Christensen, Bjorner, Kreiner, & Petersen, 2002; Fischer & Molenaar, 1995), confirmatory factor analysis (Alexander *et al*., 2017; Hattie, 1985; Rosato *et al*., 2016), or principal component analysis of the residual (Chou & Wang, 2010; Van Nes *et al*., 2011; Vaughan, 2018) can be used to confirm unidimensionality. As we will also see in the experiments, our procedure has an intrinsic drive to favour items belonging to a single dimension in the included set: including more of the same dimension helps to more reliably estimate the abilities corresponding to that dimension. The greater flexibility in fitting discrimination parameters attracts items that model different dimensions to the excluded set.

#### 3.2.3 Local independence

The responses of the items that are put to Rasch analysis are assumed to be conditionally independent of each other, given the latent variable. Dependent items may inflate scores and force the final scale's score in a particular direction (Vanhoutte *et al*., 2015), thereby increasing the risk of false positive or false negative results. The standard procedure is to keep just one of two items with highly correlated residuals. Also here, we expect our procedure to perform relatively well automatically: reliable estimation of abilities fares better from items with uncorrelated residuals than with correlated residuals.

### 3.3 Stopping criterion

The decision when the optimal combination of items to fulfil the Rasch model requirements has been achieved can be arbitrary and is at least in part based on the subjective opinion of the Rasch modeller (Bond & Fox, 2015; Robinson, Johnson, Walton, & MacDermid, 2019). Our procedure does provide an objective stopping criterion, namely at the maximum IPOQ‐LL. With the same regularization for the discrimination parameters, $\lambda_{\text{in}}=\lambda_{\text{out}}$, moving any item from the excluded to the included itemset will never decrease the IPOQ‐LL. When choosing $\lambda_{\text{in}}\gg \lambda_{\text{out}}$, as we propose, there are two counteracting effects. On the one hand, moving an item from the excluded to the included itemset tends to increase the IPOQ‐LL: it allows the abilities to adapt themselves to this item as well, increasing its corresponding log likelihood contribution. On the other hand, items in the excluded itemset enjoy more flexibility in estimating the discrimination parameter, which means that items that are best modelled with a discrimination parameter quite different from 1 may fare better in the excluded itemset.

In our experiments (data not shown, but easily reproducible), we noticed that the size of the itemset that leads to the maximum IPOQ‐LL is largely insensitive to the setting of the regularization parameters $\lambda_{\mathrm{in}}$ and $\lambda_{\mathrm{out}}$ as long as $\lambda_{\text{in}}\gg 1$ (strong regularization) and $\lambda_{\text{out}}\ll 1$ (hardly any or even no regularization).

### 3.4 Itemset selection

On top of our single criterion for the quality of any split into an included itemset $S_{\text{in}}$ and an excluded itemset $S_{\text{out}}$, we can now use any optimization procedure to determine which items to keep. In fact, almost any approach originally designed for feature subset selection can be applied here as well (Cai, Luo, Wang, & Yang, 2018; Derksen & Keselman, 1992).

For a small number of items P in the original survey, exhaustive search through all $2^{P}‐1$ combinations may still be feasible. Alternatively, we may want to first fix the size of the final questionnaire to $\left|S_{\text{in}}\right|$ and then enumerate all $\left(\begin{array}{c} \left|S_{\text{in}}\right|\\ P \end{array}\right)$ possible itemsets of that size.

For larger numbers of items in the original survey, exhaustive search is computationally too expensive and greedy approaches, such as backward elimination and forward selection, can be applied. Backward elimination is the search method of choice in standard Rasch analysis: starting from the full set of items, items are sequentially eliminated. Forward selection is the obvious alternative: starting from the empty set, items are sequentially added. In our experiments, we go for stepwise selection, which alternates between backward elimination and forward selection. Pseudocode for stepwise selection can be found in Algorithm 2.

Starting from a full itemset, $\text{OneStepBackwardElimination}\left(S_{\text{in}}\right)$ in line 5 considers all possible $\left|S_{\text{in}}\right|$ itemsets with one item fewer and returns the highest in‐plus‐out‐of‐questionnaire log likelihood as well as the itemset that leads to this maximum. $\text{OneStepForwardSelection}\left(S_{\text{in}}\right)$ in lines 8 and 11 does the same, but by taking the best of all potential itemsets that can be constructed by adding one extra item to $S_{\text{in}}$. The forward selection steps give the search procedure the possibility of (partially) recovering when backward elimination too greedily excludes items that later in the process, when fewer items are left over, may turn out to be valuable after all.

## 4 Experimental study

To evaluate our method, we conducted experiments on two artificial datasets (inhomogeneous and multi‐dimensional surveys) and three publicly available real‐world datasets. All experiments were run on a Dell PowerEdge R920, 4 × Xeon E7‐4870v2 15C 2.3 GHz processors, 3,072 GB RAM. We applied parallelization for the backward elimination function (Algorithm 3.4, line 5) and the forward selection function (Algorithm 3.4, line 8 and 11).

### 4.1 Application to artificial data

The artificial data experiments are meant to show that our semi‐automated algorithm and the standard Rasch analysis apply similar procedures for removing items (e.g., removing items which are relatively hard to predict and favouring items that belong to the same dimension). In these experiments, we consider two types of artificial surveys, which we refer to as inhomogeneous and multi‐dimensional, respectively. For both types, we create surveys with either dichotomous or polytomous (with five categories) responses. All surveys have 18 items, subdivided into three subsets of six each, and 301 subjects. Responses are generated independently from the GPCM in (1) and (2), with $m_{i}=2$ for the dichotomous case and $m_{i}=5$ for the polytomous case, and parameter settings as further detailed in Table A1.

#### 4.1.1 Inhomogeneous survey

Responses in the inhomogeneous survey are generated with different discrimination parameters $\alpha_{1‐6}\in \left\{0.04,0.045,0.05,0.055,0.06,0.065\right\}$, $\alpha_{7‐12}\in \left\{0.2,0.25,0.3,0.35,0.4,0.45\right\}$, and $\alpha_{13‐18}\in \left\{2.6,2.65,2.7,2.75,2.8,2.85\right\}$ for the first, second, and the third subsets, respectively. This makes the responses on items inhomogeneous as items in the first subset are relatively hard to predict, in the third subset relatively easy to predict, and in the second subset somewhere in between. From the goodness‐of‐fit criteria, we expect a Rasch analysis to first remove the items of the first subset, then the second subset, and finally the third subset.

**Figure 1:**
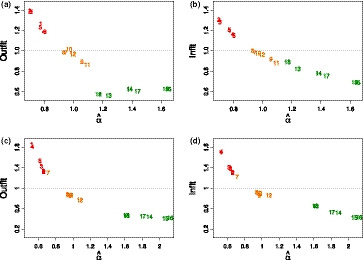
Estimated discrimination parameters ($\hat{\alpha }$) against outfit and infit of the inhomogeneous survey case: (a) $\hat{\alpha }$–outfit plot of the dichotomous test items; (b) $\hat{\alpha }$–infit plot of the dichotomous test items; (c) $\hat{\alpha }$–outfit plot of the polytomous test items; and (d) $\hat{\alpha }$–infit plot of the polytomous test items.

Figure 1 displays outfit and infit values typically used in Rasch analysis against discrimination parameter values obtained by fitting a (G)PCM to the simulated data, revealing a strong relationship between those values. This relationship applies to both the dichotomous and the polytomous case.

**Figure 2:**
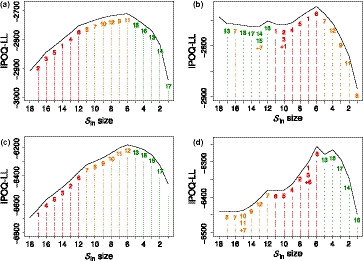
The highest IPOQ‐LL score obtained for each number of included items $\left|S_{\mathrm{in}}\right|$ when running the semi‐automated procedure on: (a) the dichotomous inhomogeneous survey; (b) the dichotomous multi‐dimensional survey; (c) the polytomous inhomogeneous survey; (d) the polytomous multi‐dimensional survey. The numbers on the plot shows in which order the items are removed; for example, in (b), $S_{\text{in}}$ size 13 was formed after removing items 14 and 15, but reintroducing item 7 which had been removed at the beginning.

Standard Rasch analysis tends to first remove the items with the highest infit and outfit values, which are those for which the responses are hardest to predict. As can be seen in Figure 2a,c, for dichotomous and polytomous test items respectively, our semi‐automated algorithm does the same for reasons explained in Section 3.2.1: *ceteris paribus*, the IPOQ‐LL score prefers items with lower discrimination parameters to be moved from the included to the excluded itemset. The maximum of the IPOQ‐LL score as a function of the number of items in the included itemset in these simulations is obtained when the six most predictive items are still included. In practice, this may fluctuate a bit, also depending on the choice of the regularization parameters $\lambda_{\mathrm{in}}$ and $\lambda_{\mathrm{out}}$.

#### 4.1.2 Multi‐dimensional survey (uncorrelated)

The multi‐dimensional survey represents a situation in which each group of items corresponds to a different dimension. To achieve this, we choose subjects' abilities $\theta_{n}=‐3,\ldots ,3$ for the first subset of items and randomly permute these for the two other subsets. The discrimination parameters for all items are the same. Which of the subsets is easiest to predict is now arbitrary, but we do expect a Rasch analysis to first remove all items from one subset, then from another subset, and only then from the remaining subset of items.

Figure 2b,d, for dichotomous and polytomous test items, respectively, show that this is indeed what happens when applying our semi‐automated procedure to the multi‐dimensional survey: the algorithm removes one item and then continues to remove items belonging to the same subset (i.e., the same dimension), before removing any item from one of the other dimensions. This is in line with our argumentation in Section 3.2.2. Which dimension is preserved in these simulations depends on the surveys' responses and cannot be argued *a priori* as for the inhomogeneous survey. Again, the maximum IPOQ‐LL occurs when six items are still included, but may slightly vary across simulations and for different choices of the regularization parameters.

#### 4.1.3 Multi‐dimensional survey (with correlations)

The correlated multi‐dimensional survey represents a situation in which each group of items corresponds to a different dimension which correlate with each other. To achieve this, we create two correlated vectors, within a range of $\left[‐4,4\right]$, for the first and the second subsets, respectively. This survey has the same setting as the previous uncorrelated multi‐dimensional survey for the discrimination and difficulty parameters, but uses only two subsets (dimensions) of polytomous test items. In this simulation, we check for five different degrees of correlation (.2, .3, .4, .5, .6).

**Figure 3:**
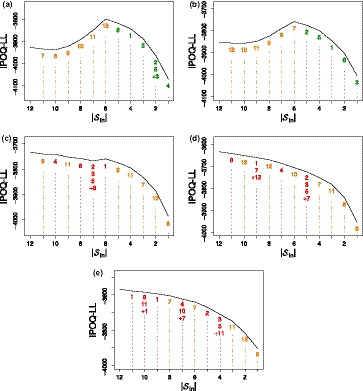
The highest IPOQ‐LL score obtained for each number of included items $\left|S_{\text{in}}\right|$ after running the semi‐automated procedure on correlated multi‐dimensional surveys with various correlations between dimensions: (a) ρ=.2; (b) ρ=.3; (c) ρ=.4; (d) ρ=.5; (e) ρ=.6.

Figure 3 shows that when the correlation is low (e.g., .2 and .3) the results are similar to the results of the uncorrelated multi‐dimensional survey (see Figure 2d). The algorithm again removes items belonging to the same subset, before removing any item from another dimension. When the correlation is higher, (e.g., ≥.4), the algorithm starts to mix, or even use all, items from both dimensions to measure the ability of the subjects. This is in line with the warning that is given in (Adams, Wilson, & Wang, 1997), that the use of a unidimensional model for a not highly correlated multidimensional scale can give biased parameter estimates.

### 4.2 Application to real‐world datasets

To validate our method on real‐world data, we searched for datasets that satisfy the following criteria.

- The original dataset (survey with responses) is publicly available.
- A manual Rasch analysis has been applied to develop an instrument.
- The final instrument contains no DIF or other special features.
- None of the authors of the current paper has been involved in the development of the instrument.
- The corresponding publication is less than 5 years old.

We found three such datasets: the Sleep Quality and Distress Inventory dataset (Morrone *et al*., 2017), the Trypophobia Questionnaire dataset (Imaizumi & Tanno, 2018) and the Coping Health Inventory for Parents instrument dataset (Gothwal, Bharani, & Reddy, 2015)..

#### 4.2.1 The Sleep Quality and Distress Inventory dataset

**Figure 4:**
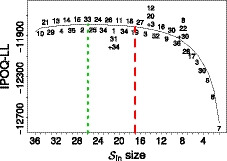
The highest IPOQ‐LL score obtained for each number of included items $\left|S_{\text{in}}\right|$ when running the semi‐automated procedure on the Sleep Quality and Distress Inventory dataset. The short‐dashed line indicates the highest IPOQ‐LL score, which is obtained when 26 items are included. The dashed line corresponds to the number of included items (17) in the original instrument, obtained through a manual Rasch procedure (Morrone *et al*., 2017). The numbers on the plot show the order in which items are removed; for example, $S_{\text{in}}$ of size 21 was formed after removing items 1 and 31, but reintroducing item 34 which had been removed before.

The Sleep Quality and Distress Inventory is an instrument that was developed to measure the effect of sleep impairment on emotional distress in patients with various sleep disorders (Morrone *et al*., 2017). The original survey consists of responses from 457 subjects to 36 polytomous questions with four response categories: `never', `sometimes', `often' and `always', coded 1 to 4, respectively (Morrone *et al*., 2017). Since the subjects rarely choose categories 3 (`often') and 4 (`always'), these categories were then combined into a new category 3, `frequently' (Morrone *et al*., 2017). Applying manual Rasch analysis, Morrone2017 ended up with a final instrument of 17 items. We will refer to this set of 17 items as the manual instrument. Using the same combination of categories, our semi‐automated procedure leads to the result shown in Figure 4. Running the whole stepwise procedure on this dataset with 36 items takes about 20 minutes. The maximum IPOQ‐LL occurs when 26 items are still included.

To prevent having to compare the 26 items from the semi‐automated procedure against the 17 items in the manual instrument, for the semi‐automated procedure we consider only the 17 items that correspond to the highest IPOQ‐LL when 17 items are still included ($\left|S_{\text{in}}\right|=17$). We will refer to this set as the semi‐automated instrument. The overlap between the semi‐automated and the manual instrument is 14 items, which can be considered large: the probability of having an overlap of 14 or more items just by chance is <$10^{‐4}$. Items 24, 31 and 34 in the manual instrument are replaced by items 22, 27 and 36 in the semi‐automated instrument (see also Table 2).

To further illustrate the clinimetric quality of both instruments, we consider standard Rasch statistics such as goodness of fit, local independence, unidimensionality and reliability. For goodness of fit and local independence we take the mean over all items. For comparison we also compute these statistics for 10,000 randomly drawn 17‐item instruments.

**Figure 5:**
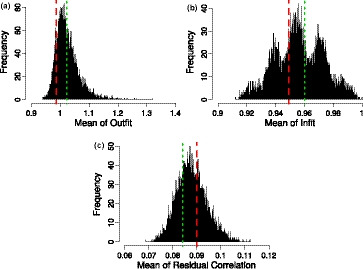
Statistics of the original 17‐item instrument from (Morrone *et al*., 2017) obtained through manual Rasch analysis (dashed line), the optimal 17‐item instrument according to the semi‐automated procedure (short‐dashed line), and 10,000 random 17‐item instruments (histogram) on the Sleep Quality and Distress Inventory dataset: (a) mean of outfit MnSq; (b) mean of infit MnSq; (c) mean of residual correlation.

Figure 5 shows histograms of some standard Rasch statistics for random instruments in which the statistics for the manual and semi‐automated instrument are marked by dashed and short‐dashed lines, respectively. The mean outfit and infit values for both instruments are pretty close to 1, the recommended value, but the same applies to (most) random 17‐item instruments. The residual correlation for the semi‐automated instrument happens to be slightly lower than that for the manual instrument, but both are well within the acceptable range, and very comparable to those for random 17‐item instruments.

Table 1 compares some of the main Rasch statistics for the semi‐automated and the manual instrument. The semi‐automated instrument has a somewhat higher IPOQ‐LL and lower mean residual correlation, where the manual instrument leads to a narrower range of the outfit and infit mean square values per item.

**Table 1:** High‐level comparison between the original 17‐item instrument from (Morrone *et al*., 2017) obtained through manual Rasch analysis and the optimal 17‐item instrument according to the semi‐automated procedure

	IPOQ‐LL	Mean Residual Correlation	Range of outfit MnSq	Range of infit MnSq
Manual	−11837	.090 (no correlation > .3)	.737–1.315	.758–1.282
Semi‐automated (no correlation > .3)	−11812	.084	.745–1.747	.570–1.307

**Figure 6:**
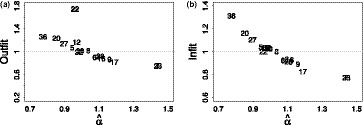
Estimated discrimination parameters ($\hat{\alpha }$) against the outfit and infit values of individual items in the semi‐automated instrument for the Sleep Quality and Distress Inventory dataset: (a) $\hat{\alpha }$–outfit plot; (b) $\hat{\alpha }$–infit plot.

Figure 6 shows the outfit and infit values of the 17 individual items in the semi‐automated instrument as a function of the estimated discrimination parameters. As expected, both the outfit and, in particular, the infit values are strongly related to the discrimination parameters. Item 22 is the exception: it has a high outfit value, yet relatively high discrimination parameter and relatively low infit value. Looking more closely, the high outfit value is solely due to a surprising response of a single subject (subject 220; see also Table 2). The high outfit value likely explains why it did not make the final manual instrument. This sensitivity of the outfit statistic to just a few unexpected responses has been pointed out by Wright and Masters (1982).

**Table 2:** Responses of subjects 6, 68 and 240 on the items in the Sleep Quality and Distress Inventory dataset that differ between the semi‐automated and manual instruments. For these subjects, it matters the most which instrument is used to estimate their abilities

Id	Semi‐automated				Manual			
	Item 22	Item 27	Item 36	Ability	Item 24	Item 31	Item 34	Ability
6	1	1	1	−4.191	1	2	2	−2.375
68	1	1	1	−4.191	1	2	1	−3
240	3	1	1	−2.338	1	1	1	−4.199

**Figure 7:**
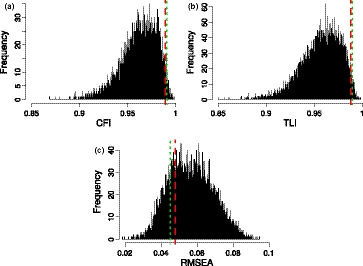
Unidimensionality test indices for the semi‐automated instrument (short‐dashed line), the manual instrument from (Morrone *et al*., 2017) (dashed line), and random 17‐item instruments on the Sleep Quality and Distress Inventory dataset: (a) comparative fit index (CFI); (b) Tucker–Lewis index (TLI); (c) root mean‐square error of approximation (RMSEA).

To evaluate the unidimensionality of the instruments we use confirmatory factor analysis. Figure 7 shows the distribution of three commonly used fit indices: the comparative fit index (CFI), Tucker–Lewis index (TLI), and root mean square error of approximation (RMSEA). For the CFI and TLI, the higher the better, with values close to 1 indicating a close fit; for RMSEA, the lower the better. Both instruments do very well with respect to CFI and TLI and perfectly fine with respect to RMSEA, in all cases well within the acceptable ranges for good unidimensionality: CFI > .95, TLI > .95, and RMSEA < .06 (Hu & Bentler, 2009).

**Figure 8:**
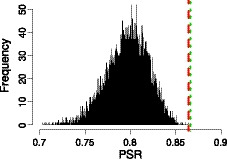
Person separation reliability (PSR) of the semi‐automated instrument (short‐dashed line), the manual instrument from (Morrone *et al*., 2017) (dashed line), and random 17‐item instruments (histogram) on the Sleep Quality and Distress Inventory dataset.

A reliability measure that is commonly used in standard Rasch analysis is person separation reliability (PSR), which indicates the overall performance of an instrument. It is the ratio of the true variance in the estimated measures to the observed variance and indicates the number of distinct person strata that can be distinguished (Wright & Masters, 1982; W. P. Fischer, 1992). This measure is comparable to Cronbach's alpha and ranges from 0 to 1, with values near to 1 indicating an excellent person separation reliability (Duncan, Bode, Lai, & Perera, 2003). The commonly used threshold for good reliability is PSR ≥ .8 (Duncan *et al*., 2003; Pesudovs, Burr, Harley, & Elliott, 2007). As shown in Figure 8, both instruments, semi‐automated and manual, excel in comparison with random instruments and have a good value of PSR, .864 and .866, respectively.

**Figure 9:**
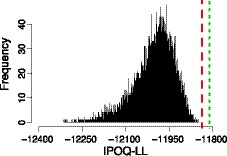
In‐plus‐out‐questionnaire log likelihood (IPOQ‐LL) values for the semi‐automated instrument (short‐dashed line), the manual instrument from (Morrone *et al*., 2017) (dashed line), and random 17‐item instruments (histogram) on the Sleep Quality and Distress Inventory dataset.

Figure 9 compares both instruments using our own IPOQ‐LL criterion. By definition, the semi‐automated instrument is very well optimized for this criterion. The manual instrument does only slightly worse and better than all randomly drawn 17‐item instruments, which appears to support our earlier argumentation that IPOQ‐LL intrinsically captures many of the properties that a typical Rasch analysis cares about.

**Figure 10:**
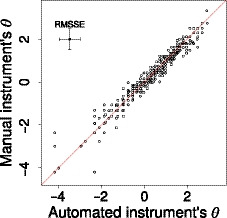
Estimated abilities for individual subjects based on the semi‐automated against those based on the manual instrument. The root mean squared standard error for the estimates on both axes is visualized through the error bars at the top left.

Considering the standard Rasch statistics, which are averages over all items and all subjects, we conclude that the manual and semi‐automated instruments are clinimetrically very similar. We expect the abilities estimated for individual subjects based on either the manual or the semi‐automated instruments to be very much alike. Figure 10 plots these estimated ability parameters for the two instruments against each other. Indeed, the estimated ability parameters for the two instruments are highly correlated (ρ=.978).

For each estimated ability, we can also compute its standard error. The root mean squared standard error, .498 and .486 for the semi‐automated and manual instruments respectively, is visualized through the error bars at the top left of Figure 10. For subjects 6, 68 and 240, corresponding to the three data points in the lower left corner away from the diagonal, the choice of instrument does appear to have a quite large effect on their estimated ability. Table 2 gives their responses on the non‐overlapping items in the two instruments. Subjects 6 and 68 reported the lowest score on the items 22, 27 and 36 which are part of the semi‐automated instrument, yet gave higher scores on items 24, 31 and 34 which are part of the manual instrument. This makes their `semi‐automated ability' considerably lower than their `manual ability'. More or less the opposite applies to subject 24, who (unexpectedly) gave the highest score on item 22, which is part of the semi‐automated instrument but not of the manual instrument.

#### 4.2.2 Trypophobia Questionnaire dataset

The Trypophobia Questionnaire is an instrument that was developed to assess subjects' feelings and somatic responses towards clusters of roughly circular objects (Imaizumi & Tanno, 2018). The original survey consists of responses from 582 subjects to 17 polytomous questions with five categories: `not at all', `slightly', `moderately', `considerably' and `extremely', coded 1 to 5, respectively (Imaizumi & Tanno, 2018).

**Figure 11:**
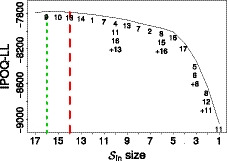
The highest IPOQ‐LL score obtained for each number of included items $\left|S_{\text{in}}\right|$ when running the semi‐automated procedure on the Trypophobia Questionnaire dataset. The short‐dashed line indicates the highest IPOQ‐LL score, which is obtained when 16 items are included. The dashed line corresponds to the number of included items (14) in the original instrument, obtained through a manual Rasch procedure (Imaizumi & Tanno, 2018). The numbers on the plot show the order in which items are removed; e.g., $S_{\text{in}}$ of size 6 was formed after removing items 8 and 15, but reintroducing item 16 which had been removed before.

According to a standard Rasch analysis, the shortened version of the Trypophobia Questionnaire (with 14 items) has slightly better psychometric properties than the full version (with 17 items) (Imaizumi & Tanno, 2018). Our semi‐automated analysis of this dataset results in Figure 11. It took about 9 minutes to search among the 17 items. In this search, the maximal IPOQ‐LL occurs when 16 items are still included, i.e., with only one item (number 9) removed. The itemset with 14 items that has the highest IPOQ‐LL is identical to the original instrument obtained through a manual Rasch analysis.

#### 4.2.3 The Coping Health Inventory for Parents Dataset

The Coping Health Inventory for Parents instrument is used to measure the coping ability of parents having children with chronic disease (Gothwal *et al*., 2015). The original survey consists of 45 items divided into three subscales, with 220 subjects. In this research we only consider the first scale about `maintaining family integration, co‐operation, and an optimistic definition of the situation', since Gothwal *et al*. (2015) analysed these scales separately using a standard Rasch analysis and found some hard‐to‐predict items in this scale.

**Figure 12:**
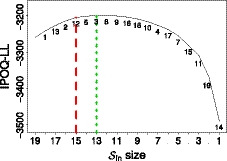
The highest IPOQ‐LL score obtained for each number of included items $\left|S_{\text{in}}\right|$ when running the semi‐automated procedure on the first subscale of the Coping Health Inventory for Parents dataset. The short‐dashed line indicates the highest IPOQ‐LL score, which is obtained when 13 items are included. The dashed line corresponds to the number of included items (15) in the original instrument, obtained through a manual Rasch procedure (Gothwal *et al*., 2015). The numbers on the plot show the in order which the items are removed; for example, $S_{\text{in}}$ of size 15 was formed after removing item 12.

The dataset of this first subscale consists of responses from 220 subjects to 19 polytomous questions with four categories: `not helpful', `minimally helpful', `moderately helpful', and `extremely helpful', coded 0 to 3, respectively. Subjects, however, rarely chose category 2 (`moderately helpful') so it was combined with category 1 (`minimally helpful') into a single new category 1 called `somewhat helpful' (Gothwal *et al*., 2015). Gothwal *et al*. (2015) concluded that the revision of the first subscale has good psychometric properties after removing four items. With the same set‐up, we applied our semi‐automated procedure and obtained the result shown in Figure 12. It took about 4 minutes to search among the 19 items. In this search, the maximum IPOQ‐LL occurs when 13 items are included. Also for this survey, the semi‐automated procedure returns the original instrument when we consider the itemset that has the maximal IPOQ‐LL for the same number of items as the original instrument (15 in this case).

## 5 Discussion and conclusion

In this paper we have described a novel procedure for semi‐automated Rasch analysis. The aim of the procedure is to optimize a new criterion, the so‐called in‐plus‐out‐of‐questionnaire log likelihood, IPOQ‐LL. The philosophy behind this criterion is that a proper instrument should not just yield reliable estimates of a subject's scores on the items that are part of the instrument, but also (albeit perhaps a bit less) on those that are left out. Through simulations and validations on real‐world data, we have shown that our semi‐automated procedure naturally incorporates desiderata for Rasch analysis related to goodness of fit and unidimensionality and leads to instruments that are very similar to or even indistinguishable from those obtained with manual Rasch analysis (when constrained to the same number of items).

In our search for a working procedure that yields results similar to standard Rasch analyses, we noticed that two ingredients are essential: flexible discrimination parameters in the generalized partial credit model (instead of a standard Rasch model with all discrimination parameters set to 1) and stronger regularization of these discrimination parameters for the included items compared to the excluded items (instead of the same or no regularization for both). Without these ingredients, the procedure has a tendency to put items that are relatively difficult to predict in the included set, so that they can play a role in the construction of the scale. By providing more flexibility to the discrimination parameters in the excluded set, items that are difficult to predict can more easily get smaller discrimination parameters, so that incorrect predictions have a smaller impact on the log likelihood. The final outcome is largely insensitive to the setting of the regularization parameters involved: sensible default settings appear to work well for all simulated and real‐world datasets considered in this paper.

A global criterion to measure the quality of any set of items, like the IPOQ‐LL introduced in this paper, can be used in conjunction with any optimization approach to find the optimal set of items. Where standard Rasch analysis effectively applies backward elimination, removing items one by one, here we applied a stepwise procedure, also allowing for items to re‐enter. At the expense of heavier computations, one can also go for (even) more involved optimization procedures such as hill climbing with restarts or evolutionary algorithms. It is doubtful, however, whether calling in heavy optimization machinery really pays off in practice: even though simpler optimization approaches may fail to find the global optimum and hence the `best' instrument according to the precise optimization criterion, instruments corresponding to a local optimum may well be clinimetrically very similar. More generally, considering all the uncertainties involved, due to the finite number of subjects in any dataset, the vagueness of optimality criteria, and the arbitrariness in the statistical models applied, it hardly makes sense to claim that a procedure, be it manual or (semi‐)automated, leads to an incontestable optimal instrument. As also suggested by the histograms in Figures 5, 6, 7, 8, 9 and the comparison between the manual and semi‐automated instrument in Section 4.2.1, if a procedure finds one `optimal' instrument, there are likely many more that are virtually indistinguishable.

Our procedure can be extended in various ways, for example by adapting the underlying statistical model. An obvious and important extension would be the detection of differential item functioning, which occurs when individuals from different groups (e.g., male versus female) but with the same apparent ability systematically respond differently to a particular item. Many methods and procedures have been developed to detect DIF (Holland & Thayer, 1986; Komboz, Strobl, & Zeileis, 2018; Lord, 2012; Magis & Tuerlinckx, 2015; Schauberger & Tutz, 2016; Swaminathan & Rogers, 1990; Tutz & Schauberger, 2015). In our framework, DIF detection may be implemented by extending the GPCM to include a group‐dependent term, for example, along the lines of Schauberger and Mair (2019). Another relevant extension would be to allow for missing values under different assumptions, which is an active research area on its own (Thomas, Schmidt, Erbacher, & Bergeman, 2016).

Another potential extension could be to try and derive multi‐dimensional scales from a single dataset. On the multi‐dimensional surveys of Section 4.1.2, 4.1.3, and Appendix A1.2, a greedy sequential approach is likely to work: apply the semi‐automated procedure to find an instrument, remove the corresponding items from the original dataset, and apply the same procedure once more to find the next instrument. A more involved integrated approach would adapt the GPCM to allow for multiple abilities per subject, corresponding to different dimensions, for example along the lines of Liu, Magnus, O'Connor, and Thissen (2018), Adams *et al*. (1997) and Kelderman (1996).

With or without extensions, we are careful to frame the procedure as semi‐automated rather than fully automated. First of all, what comes out of the Rasch analyses, whether it is manual or semi‐automated, will always depend on what goes in. So especially for creating a preliminary questionnaire and dataset, clinical expertise is required to ask the right questions. Furthermore, in subsequent analyses, one cannot do without sanity checks, much as in any statistical analysis. These include, for example, checks for underutilized categories, rescoring of items with disordered thresholds, as well as various after‐run analyses. Given an appropriate original survey and accompanied with necessary sanity checks, our procedure has the potential to develop a valid, reliable and clinimetrically robust instrument in a less time‐consuming and more objective manner, thereby challenging the current practice of Rasch analyses and questioning the need for cumbersome manual procedures.

## Author contributions

**Feri Wijayanto** (Conceptualization; Investigation; Methodology; Software; Visualization; Writing – original draft; Writing – review & editing) **Karlien Mul** (Conceptualization; Data curation; Validation; Writing – original draft; Writing – review & editing) **Perry Groot** (Conceptualization; Formal analysis; Methodology; Supervision; Visualization; Writing – original draft; Writing – review & editing) **Baziel G.M. van Engelen** (Conceptualization; Supervision; Validation; Writing – original draft; Writing – review & editing) **Tom Heskes** (Conceptualization; Formal analysis; Methodology; Supervision; Visualization; Writing – original draft; Writing – review & editing).

## Conflicts of interest

All authors declare no conflict of interest.

## Figures and Tables

**Figure A1:**
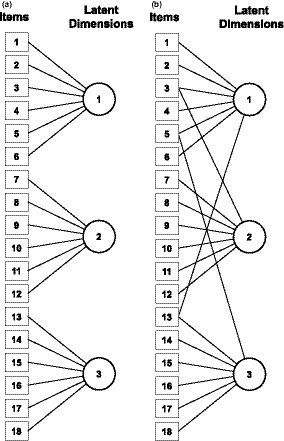
Multidimensionality at the item level: (a) between‐item model; (b) within‐item model

**Figure A2:**
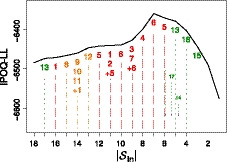
The highest IPOQ‐LL score obtained for each number of included items $\left|S_{\text{in}}\right|$ when running the semi‐automated procedure on the within‐item multidimensional survey dataset. The numbers on the plot show the order in which items are removed; for example, $S_{\text{in}}$ of size 11 was formed after removing items 1 and 2, but reintroducing item 5 which had been removed before

**Figure A2:**
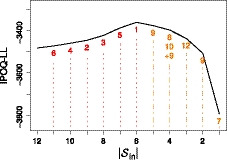
The highest IPOQ‐LL score obtained for each number of included items $\left|S_{\text{in}}\right|$ when running the semi‐automated procedure on surveys with two different local item dependence effects, $\sigma^{2}=0$ and $\sigma^{2}=4$ for subset 1 and subset 2, respectively. The numbers on the plot show the order in which items are removed; fore example, $S_{\text{in}}$ of size 4 was formed after removing items 8 and 10, but reintroducing item 9 which had been removed before.

## Data Availability

The data that support the findings of this study are openly available in figshare at https://doi.org/10.1371/journal.pone.0118189.s001, within the publication of: Gothwal *et al*. (2015) Additional file of the publication of: Imaizumi and Tanno (2018) figshare at https://doi.org/10.1371/journal.pone.0180743.s002, within the publication of: Morrone *et al*. (2017).

## Footnotes

^1^ See https://www.rasch.org/software.htm

^2^
https://github.com/fwijayanto/autoRasch

## Appendix A1 More information on artificial dataset simulation

### A.1.1 Artificial dataset parameters

This appendix describes how the artificial datasets for the experiments in Section 4.1 are generated (see Table A1).

**Table A1:** Artificial datasets specification

Parameters	Inhomogeneous survey			Multi‐dimensional survey (uncorrelated)			Multi‐dimensional survey (with correlation)	
Item numbering	Subset 1 (1–6)	Subset 2 (7–12)	Subset 3 (13–18)	Subset 1 (1–6)	Subset 2 (7–12)	Subset 3 (13–18)	Subset 1 (1–6)	Subset 2 (7–12)
Dichotomous								
$\left\{\theta_{n}\right\}$	${\left\{0.02\left(n‐1\right)‐3\right\}}_{n=1}^{301}$			${\left\{0.02\left(n‐1\right)‐3\right\}}_{n=1}^{301}$	Permutation of θ in subset 1	Permutation of θ in subset 1	–	
$\left\{\beta_{i}\right\}$	${\left\{i‐3.5\right\}}_{i=1}^{6}$			${\left\{i‐3.5\right\}}_{i=1}^{6}$				
$\left\{\alpha_{i}\right\}$	${\left\{0.005i+0.035\right\}}_{i=1}^{6}$	${\left\{0.05i+0.15\right\}}_{i=1}^{6}$	${\left\{0.05i+2.55\right\}}_{i=1}^{6}$	1				
Polytomous								
$\left\{\theta_{n}\right\}$	${\left\{0.02\left(n‐1\right)‐3\right\}}_{n=1}^{301}$			${\left\{0.02\left(n‐1\right)‐3\right\}}_{n=1}^{301}$	Permutation of θ in subset 1	Permutation of θ in subset 1	Two correlated vectors $\rho =\left\{.2,.3,.4,.5,.6\right\}$	
$\left\{\beta_{\mathrm{ij}}\right\}$	${\left\{\left(‐3.7+0.5i\right)‐\left({\left\{0.8\left(j‐1\right)\right\}}_{j=1}^{4}\right)\right\}}_{i=1}^{6}$			${\left\{\left(‐3.7+0.5i\right)‐\left({\left\{0.8\left(j‐1\right)\right\}}_{j=1}^{4}\right)\right\}}_{i=1}^{6}$			${\left\{\left(‐3.7+0.5i\right)‐\left({\left\{0.8\left(j‐1\right)\right\}}_{j=1}^{4}\right)\right\}}_{i=1}^{6}$	
$\left\{\alpha_{i}\right\}$	${\left\{0.005i+0.035\right\}}_{i=1}^{6}$	${\left\{0.05i+0.15\right\}}_{i=1}^{6}$	${\left\{0.05i+2.55\right\}}_{i=1}^{6}$	1			1	

#### A1.2 Within‐item multi‐dimensional survey

In Section 4.1.2 each item only relates to one dimension, whereas in the within‐item multi‐dimensional survey each item relates to more than one dimension. Figure A1 shows the difference between the within‐item and between‐item multi‐dimensional models. In the within‐item multi‐dimensional case, the membership of the groups becomes less clear and there are items that belong to more than one group. In order to achieve this, we choose subjects' abilities $\theta_{n}=‐3,\ldots ,3$ and item difficulties $\beta_{i}=‐2.5,\ldots ,2.5$ as in Section 4.1.2. Instead of applying the structure as in A1a, however, we apply the structure in Figure A1b. In this simulation, we only create surveys with polytomous (five categories of) responses. Responses are generated from the multidimensional random coefficients multinomial logit model as in Adams *et al*. (1997).

Figure A2 shows that applying the algorithm to the within‐item multi‐dimensional survey will lead to a similar result. The maximum IPOQ‐LL occurs when seven items from the third dimension are included $\left\{5,13,14,15,16,17,18\right\}$. Yet, again, from which dimension the items are preserved in this simulation depends on the survey responses and cannot be argued *a priori*

### A1.3 Survey with local item dependence effect

This survey represents a situation in which each group of items corresponds to a different testlet (item bundle). Each testlet has a different variance effect that corresponds to the local item dependence effect. To achieve this, similarly to the multi‐dimensional surveys, we choose subjects' abilities $\theta_{n}=‐3,\ldots ,3$ and item difficulties $\beta_{i}=‐2.5,\ldots ,2.5$ for all subsets of items. To simulate the local dependence effect of the testlets, we generate two subsets with $\sigma^{2}=0$ and $\sigma^{2}=4$ for subset 1 and 2, respectively. A larger variance means larger interaction between persons and items (local item dependence) within the testlet (Wainer & Wang, 2000; Wang & Wilson, 2005). All of the responses are generated independently from the Rasch testlet model in Wang and Wilson (2005).

Figure A3 shows that our algorithm will remove items from subset 1 and favour subset 2, the subset with the larger item dependence effect. In contrast, standard Rasch analysis is to likely remove items from subset 2 since they have a larger amount of local item dependence (e.g., a higher residual correlation among items within subset 2) than those from subset 1. After completely removing items from subset 1, however, there is no evidence of local item dependence within subset 2 due to the low correlation of the residual. Therefore, we would argue that it is better to keep the items from subset 2 since the resulting responses give a better separation between subjects' abilities
